# The Potential Effect of Fucoidan on Inhibiting Epithelial-to-Mesenchymal Transition, Proliferation, and Increase in Apoptosis for Endometriosis Treatment: In Vivo and In Vitro Study

**DOI:** 10.3390/biomedicines8110528

**Published:** 2020-11-22

**Authors:** Li-Chun Chang, Yi-Fen Chiang, Hsin-Yuan Chen, Yun-Ju Huang, An-Chieh Liu, Shih-Min Hsia

**Affiliations:** 1 School of Nutrition and Health Sciences, College of Nutrition, Taipei Medical University, Taipei 11031, Taiwan; a37130763@gmail.com (L.-C.C.); yvonne840828@gmail.com (Y.-F.C.); hsin246@gmail.com (H.-Y.C.); d04641004@ntu.edu.tw (Y.-J.H.); 2 Department of Obstetrics and Gynecology, Taipei Medical University Hospital, Taipei 11031, Taiwan; 918048@h.tmu.edu.tw; 3 Graduate Institute of Metabolism and Obesity Sciences, College of Nutrition, Taipei Medical University, Taipei 11031, Taiwan; 4 School of Food and Safety, Taipei Medical University, Taipei 11031, Taiwan; 5 Nutrition Research Center, Taipei Medical University Hospital, Taipei 11031, Taiwan

**Keywords:** endometriosis, fucoidan, epithelial–mesenchymal transition, inflammatory, vascular endothelial growth factor

## Abstract

Endometriosis is common in reproductive-age women and its pathology is to increase proliferation and migration to enhance epithelial-to-mesenchymal transition progression (EMT). However, treatments are currently limited, so it is important to explore new therapeutic drugs. Hence, in this study, we investigate the therapeutic effect of fucoidan (FC) on the progression and mechanisms of endometriosis. The cell viability of endometrial cell lines End1/E6E7 and Vk2/E6E7 treated with different concentrations of FC were assessed by 3-(4,5-Dimethylthiazol-2-yl)-2,5-diphenyltetrazolium bromide (MTT) assay and cell counting. Cell migration was evaluated using wound-healing assay. In an in vivo experiment, female Balb/c mice received surgically induced endometriosis followed by different concentrations of fucoidan for 6 weeks. High-frequency ultrasound imaging was applied to detect subsequent lesion growth. The results demonstrated that fucoidan inhibited the viability and migration ability of End1/E6E7 and Vk2/E6E7 cells. Additionally, the administration of fucoidan reduced the volume and weight of endometriotic lesions, decreased inflammatory cytokines and vascular endothelial growth factor (VEGF) of serum and lesions, and improved EMT proliferation and apoptosis-related protein expression. For the first time, fucoidan indicated anti-proliferative and anti-inflammatory effects as well as inhibited EMT progression and induced apoptosis, improving endometriosis.

## 1. Introduction

Endometriosis is a chronic, estrogen-dependent benign inflammatory disease that occurs in reproductive-age women and affects 6–10% of women [1,2]. It presents several severe clinical features, including chronic pelvic pain, dysmenorrhea, dysuria, and infertility [3,4,5]. It has also been shown that endometriosis increases the risk of gynecologic cancers, such as ovarian cancer and breast cancer [6]. However, the endometriosis pathogenesis has not been cleared, although retrograde menstruation has recently become an extensively accepted theory. During menstruation, endometrial tissue in the menstrual blood flows back through the fallopian tube and into the ovary or peritoneal cavity, thereby adhering to other tissues and growing [7,8,9].

The factors of endometriosis progression include proliferation, angiogenesis, and epithelial–mesenchymal transition (EMT). Estrogen may play a key role in endometriosis progression [10] and may induce EMT, inflammation, and angiogenesis through estrogen receptor α (ER-α) [11]. Estrogen works through the estrogen receptors, including ER-α and ER-β, which present in different tissue [12]. ERα is present mainly in the uterus, mammary gland, and adipose tissue. While ER-β is mainly in the prostate, colon, and immune system [13]. The reduction of endometriosis may alter the expression of ER-α [14,15]. The higher expression of ER is also present in the endometriosis tissue and, specifically, there is a higher ER-β/ ER-α ratio than in normal endometrium [15]. The higher expression and ratio of ER may contribute to the severity, proliferation, inflammation, and apoptosis inhibition of endometriosis [12].

In endometriosis patients, macrophages are significantly elevated in the peritoneal fluid and eutopic endometrium [16,17]. Through macrophage or directly, estrogen can induce endometriotic-cell-secreted pro-inflammatory cytokines like tumor necrosis factor-α (TNF-α), interleukin-1β (IL-1β), and interleukin-6 (IL-6). When the inflammatory factor increases, the ability of anti-apoptosis, proliferation, invasion, and the migration ability is elevated in endometriosis cells [18]. In addition, TNF-α and IL-1β could induce NF-κB expression and further regulate vascular endothelial growth factor (VEGF) expression. Increased vascular endothelial growth factor (VEGF) expression could also induce angiogenesis and EMT [18,19,20].

EMT plays a critical role in endometriosis by promoting invasion and the migration ability [21,22]. The indicator of EMT is the reduction in E-cadherin and the increase in N-cadherin [23]. When EMT occurs, the cell–cell adhesion leaks to increase cell migration and invasion and elevates the anti-apoptosis ability of endometriotic cells [24]. The EMT-related proteins Snail and Slug can reduce N-cadherin and Vimentin and increase E-cadherin protein expression to increase EMT progression [25]. A previous study indicated that estrogen may induce Snail and Slug protein expression to promote EMT progression [20].

The treatments of endometriosis are mainly used in medical therapy to manage hormone secretion and reduce pain, which still exists as a side effect or limited benefit [26]. Natural products have the potential of anti-proliferation and anti-inflammatory effects, can reduce the progression of endometriosis, and have less side effects [27]. Fucoidan (FC) is found in brown seaweed and is a sulfated fucose-rich polysaccharide. A previous study indicated that FC has antioxidant, antitumor, anti-inflammatory, and anti-angiogenesis abilities [28,29,30,31], showing its potential for anti-endometriosis progression. However, the effects of FC on endometriosis are still unclear. Therefore, the purpose of this study is to investigate FC’s effect as a potential therapeutic compound to ameliorate endometriosis.

## 2. Materials and Methods

### 2.1. Reagents

Oligo-Fucoidan was obtained from Hi-Q Marine Biotech International Ltd. (New Taipei City, Taiwan), and 17β-estradiol (E2) was supplied and purchased from Sigma-Aldrich (St. Louis, MO, USA). Keratinocyte serum-free medium, human recombinant epidermal growth factor (EGF; 0.1 ng/mL), and bovine pituitary extract (0.05 mg/mL) were purchased from Thermo Fisher Scientific (Waltham, MA, USA). Trypan blue and a bicinchoninic acid (BCA) protein assay kit were purchased from T-Pro Biotechnology (Dublin, UK). 3-(4,5-Dimethylthiazol-2-yl)-2,5-diphenyltetrazolium bromide (MTT) (catalog number: ab146345) was purchased from Abcam (Cambridge, MA, USA).

### 2.2. Cell Culture

Human endometriosis cell lines End1/E6E7 and Vk2/E6E7 were purchased from the American Type Culture Collection (ATCC, Manassas, VA, USA) and cultured in keratinocyte serum free medium (Gibco, Waltham, MA, USA) with bovine pituitary extract (0.05 mg/mL), human recombinant epidermal growth factor (EGF; 0.1 ng/mL), calcium chloride (44.1 mg/L), and 1% antibiotics (CORNING, Manassas, VA, USA, 10,000 unit/mL penicillin, 10,000 μg/mL streptomycin, 25 μg/mL amphotericin with 8.5 g/L NaCl) at 37 °C in an incubator with an atmosphere of 5% CO_2_.

### 2.3. Cell Viability Assay

Cell viability assays were performed using 3-(4,5-Dimethylthiazol-2-yl)-2,5-diphenyltetrazolium bromide (MTT) assay. End1/E6E7 and Vk2/E6E7 (1.5 × 10^4^ cells per well) were seeded in 96-well plates for 24 h and then treated with E2 (0.01, 0.1, 1, 10, 100 nM), FC (0.25, 0.5, 1 mg/mL) or quercetin (5, 10, 20, 50 μM) (positive control) [32] in fresh medium for 48 h. The MTT diluted with fresh medium (1 mg/mL) was added to each well for 3 h, and then absorbance was measured at 570 nm with a reference wavelength of 630 nm by using VERSA Max microplate reader (Molecular Devices, San Jose, CA, USA).

### 2.4. Cell Counting Assay

Cell counting assays were performed using trypan blue exclusion assay. End1/E6E7 (5 × 10^5^ cells per well) and Vk2/E6E7 (3 × 10^5^ cells per well) were seeded in 6-well plates for 24 h and then treated with E2 (1 nM) and FC (0.25, 0.5, 1 mg/mL) or quercetin (20 μM) in fresh medium for 48 h. Trypsin-EDTA solution was used to harvest the cells from 6-well plates. Next, the cells and solution were collected, and trypan blue staining was used to determine the number of cells. Hemocytometer was used to calculate the total number of cells.

### 2.5. Wound Healing Assay

End1/E6E7 (3 × 10^5^ cells per well) and Vk2/E6E7 (2 × 10^5^ cells per well) were seeded in 24-well plates for 24 h. After 24 h, the endometriosis cells monolayer was scraped with a sterile micropipette tip and then treated with E2 (100 nM) and FC (0.25, 0.5, 1 mg/mL) or quercetin (20 μM) in fresh medium for 8 h. The wound closure was photographed and analyzed at 0 and 8 h. The gap area of the wound was measured by Image J software (NIH, Bethesda, MD, USA), and the data were normalized to the average of the control group.

### 2.6. Western Blot Analysis

Cell and lesion lysates were prepared in radioimmunoprecipitation assay (RIPA) with protease inhibitor and phosphatase inhibitor (Roche, Mannheim, Baden-Württemberg, Germany). Protein quantity was evaluated by BCA protein assay. A sample of cell and lesion lysates (30 μg total protein) was loaded onto 8–12% SDS-polyacrylamide gels and transferred to polyvinylidene fluoride (PVDF) membrane and exposed to primary antibodies, including anti-E-cadherin (1:200; Santa Cruz Biotechnology, Heidelberg, Germany), anti-N-cadherin (1:200; Santa Cruz Biotechnology, Heidelberg, Germany), anti-Vimentin (1:1000; Santa Cruz Biotechnology, Heidelberg, Germany), anti-Snail (1:1000; Santa Cruz Biotechnology, Heidelberg, Germany), anti-Slug (1:500; Santa Cruz Biotechnology, Heidelberg, Germany), anti-Bax (1:1000; Cell Signaling Technology, Beverly, MA, USA), anti-Bcl-2 (1:1000; Santa Cruz Biotechnology, Heidelberg, Germany), anti-ER-α (1:1000; Proteintech, IL, USA), anti-PCNA (1:1000; Cell Signaling Technology, Beverly, MA, USA), GAPDH (1:10,000; Proteintech, Rosemont, IL, USA), and β-actin (1:1000; Santa Cruz Biotechnology, Heidelberg, Germany) for 24 h at 4 °C. The membranes were subsequently incubated with an HRP-conjugated secondary antibody (1:10,000) for 1 h at room temperature. Then, the bands were detected using enhanced chemiluminescence (ECL, T-Pro Biotechnology, Dublin, UK). The Western blot bands were quantified using ImageJ software (National Institutes of Health, Bethesda, MD, USA). Results were corrected by GAPDH and β-actin to normalize the loading.

### 2.7. Animals

Mature (6-week-old) female Balb/c mice were purchased from BioLASCO (Taipei, Taiwan) and allowed to acclimate to the environment for two weeks prior to surgery. Mice were maintained in a barrier unit in a well-controlled, pathogen-free environment with regulated cycles of 12 h light/12 h dark (22–25 °C). Mice had free access to food and water. All experiment processes were performed according to the protocols approved (21 August 2019) by the Institutional Animal Care and Use Committee (IACUC) of Taipei Medical University (IACUC Approval No. LAC-2019-0259).

### 2.8. Mouse Model for Endometriosis

A mouse model of endometriosis was used as previously described [33]. Using Zoletil and Rompun (1:1 mixed) anesthesia to anesthetize mice by intraperitoneal injection (1 mL/kg bodyweight (BW)). After anesthesia, we conducted oophorectomy and cut the left uterine horn. The tissue was cut into two equal-sized pieces by biopsy punch (2.0 mm). Each piece was implanted onto both sides of the peritoneum using a 6-0 black silk suture. For the sham group, the fat pad was implanted onto both sides of the peritoneum. After surgery, ibuprofen 3 mg/100 g BW was administered by intraperitoneal injection. Estradiol 10 mg/kg BW was administered subcutaneously twice a week throughout the experiment.

On day 14, 42 mice were randomized into seven groups according to the size of endometriotic lesions between each group, including the control, sham, E2, low dose of fucoidan (LFC) (10 mg/kg BW), mid dose of fucoidan (MFC) (50 mg/kg BW), high dose of fucoidan (HFC) (150 mg/kg BW), and quercetin (100 mg/kg BW). Administration was by oral gavage for 42 days. The growth of endometriosis lesions was subsequently analyzed by high-resolution ultrasound imaging (Prospect 3, S-Sharp) every week, which has been proven as a useful approach in the diagnosis of endometriosis [34]. At the end of the experiment, the heart blood was collected for the analysis of the mice, and the endometriotic lesions’ volume and weight were measured by Vernier caliper and scale.

### 2.9. Quantification of Cytokines

Interleukin 1β (IL-1β) (MLB00C, R&D, Minneapolis, MN, USA), tumor necrosis factor-α (TNF-α) (430907, Biolegend, San Diego, CA, USA), vascular endothelial growth factor (VEGF) (MMV00, R&D, Minneapolis, MN, USA) in the blood serum, and VEGF in endometriosis lesions were quantified by enzyme-linked immunosorbent assay (ELISA) kit following the manufacturer’s protocol.

### 2.10. Statistical Analysis

Results are shown as means ± SD or SEM. The GraphPad Prism version 5.01 (GraphPad Software, San Diego, CA, USA) was used for statistical analysis. Statistical comparisons between two groups were made by Student’s *t*-test. Comparisons between more than two groups were made by one-way analysis of variance (ANOVA), followed by Tukey’s test. *p*-values of < 0.05 were considered significant.

## 3. Results

### 3.1. Effects of FC on β-Estradiol (E2)-Induced Proliferation of Endometriosis Cells

**Figure 1:**
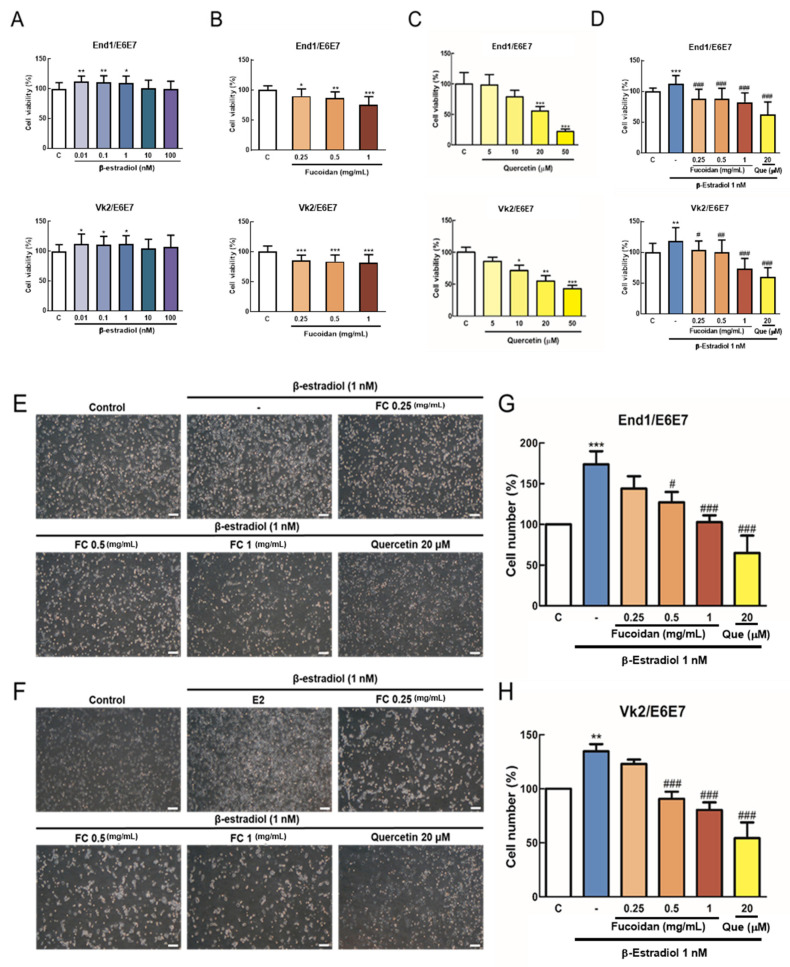
(**A**) Cell proliferation analysis in End1/E6E7 and Vk2/E6E7 cells treated with β-estradiol (*n* = 4). (**B**) Cell proliferation analysis in End1/E6E7 and Vk2/E6E7 cells treated with fucoidan (FC) (*n* = 3). (**C**) Cell proliferation analysis in End1/E6E7 and Vk2/E6E7 cells treated with quercetin (Que) (*n* = 3). (**D**) Cell proliferation analysis in End1/E6E7 and Vk2/E6E7 cells treated with β-estradiol, FC, and Que (*n* = 3). (**E**, **F**) The morphology of End1/E6E7 and Vk2/E6E7 cells treated with β-estradiol, FC, and Que. (**G**,**H**) The cell numbers of End1/E6E7 and Vk2/E6E7 cells treated with β-estradiol, FC, and Que (*n* = 3). Images were observed under a microscope at ×100 magnification. * *p* < 0.05, ** *p* < 0.01, *** *p* < 0.001 compared with the control group, # *p* < 0.05, ## *p* < 0.01, ### *p* < 0.001 compared with E2 group. Scale bar: 20 μm.

3-(4,5-Dimethylthiazol-2-yl)-2,5-diphenyltetrazolium bromide (MTT) assay was used to evaluate the viability change. E2 (0.01, 0.1, 1 nM) treatment significantly induced the growth of End1/E6E7 and Vk2/E6E7 cells (Figure 1A). FC (0.25, 0.5, 1 mg/mL) treatment decreased the growth of End1/E6E7 and Vk2/E6E7 cells (Figure 1B). To investigate the effects of FC on E2-induced proliferation of End1/E6E7 and Vk2/E6E7 cells, combination treatment was compared with E2 (1 nM). The results show that FC inhibits E2-induced proliferation of End1/E6E7 and Vk2/E6E7 cells (Figure 1D). Through the cell counting, we also confirmed that FC statistically decreased the proliferation of End1/E6E7 and Vk2/E6E7 cells induced by E2 (Figure 1G,H).

### 3.2. Effects of FC on β-Estradiol-Induced Migration and EMT-Related Protein Expression of Endometriotic Cells

**Figure 2:**
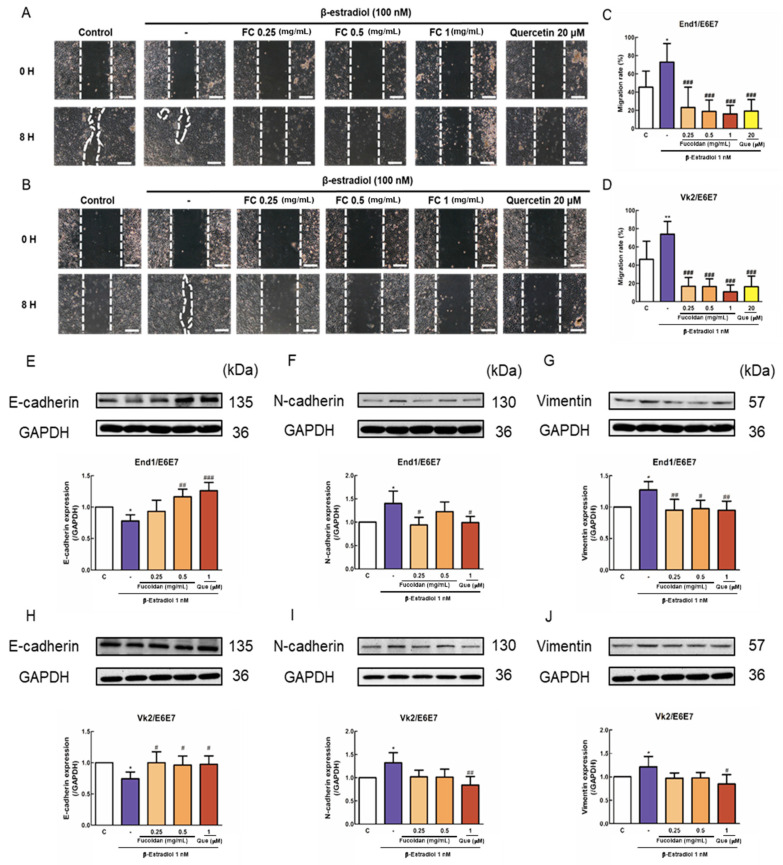
End1/E6E7 and Vk2/E6E7 cells were allowed to grow for 8 h following treatment with β-estradiol (100 nM) with or without fucoidan (0.25–1 mg/mL). (**A**,**B**) The wound healing image after 0 H and 8 H treatments (**C**,**D**) The migration image and rate of End1/E6E7 and Vk2/E6E7. (**E**–**J**) Expression of E-cadherin, N-cadherin, and Vimentin in End1/E6E7 and Vk2/E6E7 cells treated with FC and β-estradiol. The values of the band intensity represent the densitometric estimation of each band normalized to GAPDH (*n* = 4–6). Images were observed under a microscope at ×100 magnification. * *p* < 0.05, ** *p* < 0.01 compared with the control group, # *p* < 0.05, ## *p* < 0.01, ### *p* < 0.001 compared with the E2 group. Scale bar: 20 μm.

By using wound healing assay, FC (0.25, 0.5, 1 mg/mL) significantly reduced E2 (100 nM) and induced the migration rates of End/E6E7 and Vk2/E6E7 cells (Figure 2C,D).

To explore the mechanisms of inhibition in cell migration by FC, the expression of proteins regulating migration progression was analyzed by Western blot. E2 (100 nM) treatment significantly increased N-cadherin and Vimentin and reduced E-cadherin protein expression. In contrast, FC significantly reduced N-cadherin and Vimentin and increased E-cadherin protein expression in End1/E6E7 and Vk2/E6E7 cells (Figure 2E–J). Together, the results imply that FC inhibited E2-induced EMT of End1/E6E7 and Vk2/E6E7 cells.

### 3.3. Effects of FC on Endometriotic Lesions Growth in In Vivo Study

**Figure 3:**
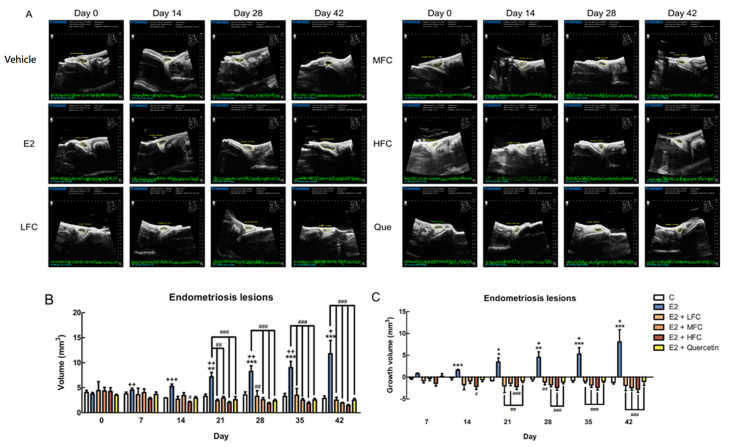
The endometriotic lesions of animals that received vehicle, β-estradiol (E2), low dose of fucoidan (LFC, 10 mg/kg), mid dose of fucoidan (MFC, 50 mg/kg), high dose of fucoidan (HFC, 150 mg/kg), and quercetin (Que, 100 mg/kg) for 42 days. (**A**) The image of endometriotic lesion and (**B**,**C**) Growth of endometriotic lesions for 42 days (*n* = 4–10). * *p* < 0.05, ** *p* < 0.01, *** *p* < 0.001 compared with the control group, # *p* < 0.05, ## *p* < 0.01 ### *p* < 0.001 compared with E2 group, + *p* < 0.05, ++ *p* < 0.01, +++ *p* < 0.001 compared with day 0.

**Figure 4:**
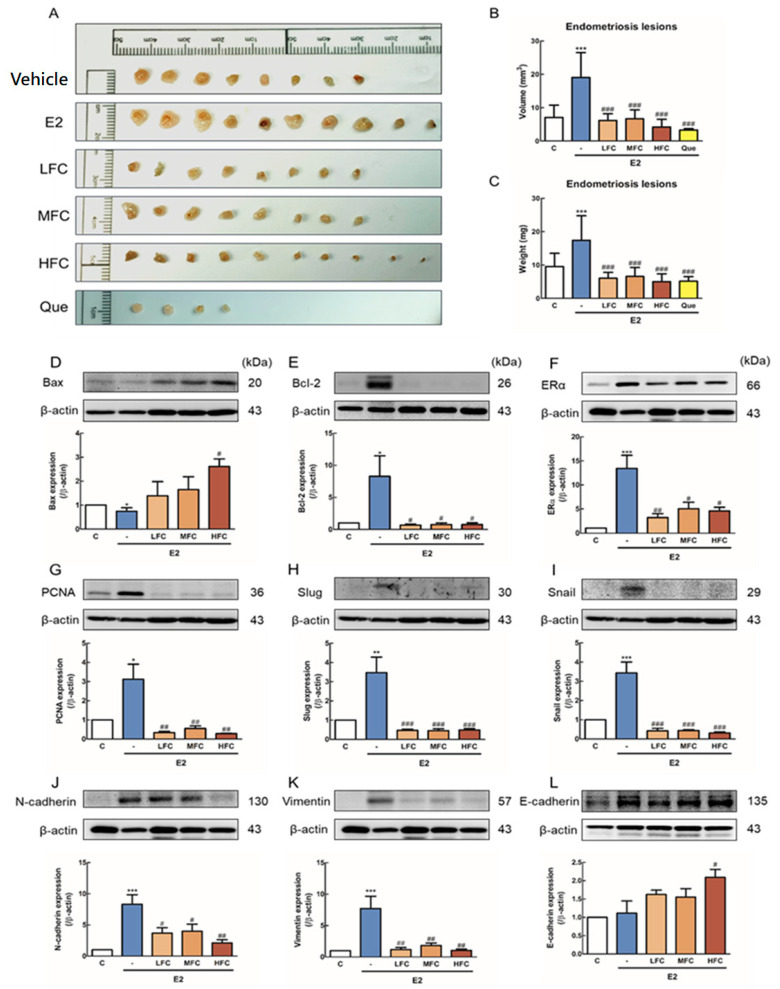
The endometriotic lesions of animals that received vehicle, β-estradiol (E2), low dose of fucoidan (LFC, 10 mg/kg), mid dose of fucoidan (MFC, 50 mg/kg), high dose of fucoidan (HFC, 150 mg/kg), and quercetin (Que, 100 mg/kg). (**A**) The image of endometriotic lesions (**B**,**C**) Volume and weight of endometriotic lesions (*n* = 4–10). (**D**–**L**) Expression of Bax (**D**), Bcl-2 (**E**), estrogen receptor -α (ER-α) (**F**), PCNA (**G**), Slug (**H**), Snail (**I**), N-cadherin (**J**), Vimentin (**K**), and N-cadherin (**L**) in endometriotic lesions. The values of the band intensity represent the densitometric estimation of each band normalized to β-actin (*n* = 3–5). * *p* < 0.05, ** *p* < 0.01, *** *p* < 0.001 compared with the control group, # *p* < 0.05, ## *p* < 0.01, ### *p* < 0.001 compared with the E2 group.

We established an experimental endometriosis mouse model to verify the anti-proliferation and apoptotic effects of FC in an in vivo study. The volume of endometriotic lesions decreased in the LHC-, MFC-, and HFC-treated groups compared with E2-treated mice, as shown by ultrasound (Figure 3A). Quantitative data analysis found that the endometriotic lesion volumes of the LFC, MFC, and HFC groups were significantly smaller than those of the E2 group (Figure 3B). Furthermore, the endometriosis lesion growth volume of the LFC, MFC, and HFC groups were also smaller than that of the E2 group (Figure 3C). Similarly, after the mice were sacrificed and endometriotic lesions were collected, it was found that the volume and weight of the LFC, MFC, and HFC groups were significantly lower than that of the E2 group (Figure 4B,C).

### 3.4. Effects of FC on Apoptosis, Proliferation-Related Protein, and ER-α Expression of endometriotic Lesions in Mice

We next investigated the apoptotic effects of FC on endometriotic lesions in mice by Western blot. The protein expression was significantly reduced in Bax and significantly increased in Bcl-2, ER-α, and PCNA of the E2 group compared with that of the control group (Figure 4D–G). However, the expression of Bax was effectively increased in the HFC group, and the Bcl-2, ER-α, and PCNA were significantly decreased in the LFC, MFC, and HFC groups when compared with the E2 group (Figure 4D–G). This shows that FC can significantly activate apoptosis and proliferation progression by affecting ER-α expression.

### 3.5. Effects of FC on EMT-Related Protein of Endometriotic Lesions in Mice

Since EMT plays a critical role in endometriosis, which affects proliferation, apoptosis, and migration, we investigated the expression of EMT-related proteins of endometriosis lesions in mice. The protein expression was significantly increased in Snail, Slug, N-cadherin, and Vimentin of the E2 group compared with the control group (Figure 4H–K). However, the expression of Snail, Slug, N-cadherin, and Vimentin were significantly reduced in the FC-administered group (LFC, MFC, HFC group) compared to the E2 group. Furthermore, the increase in E-cadherin was more effective in the HFC group than in the E2 group (Figure 4H–L).

### 3.6. Effects of FC on VEGF, TNF-α, and IL-1β in Endometriotic Mice

**Figure 5:**
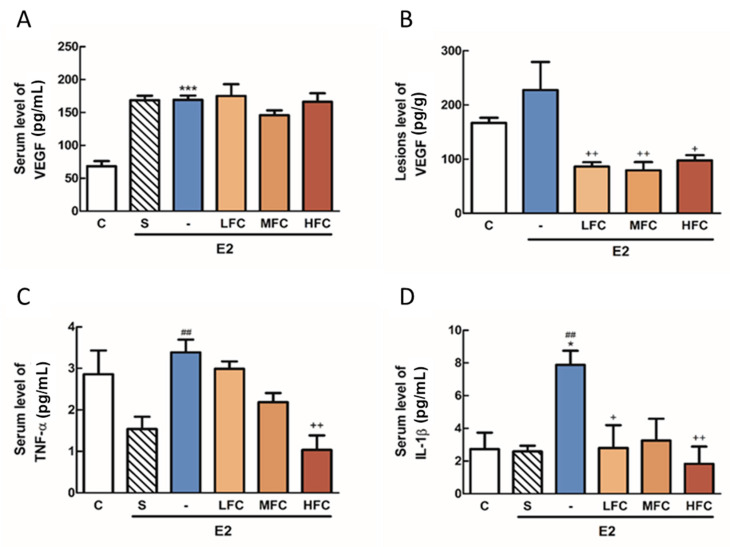
The serum levels of vascular endothelial growth factor (VEGF) (*n* = 4–7) (**A**), TNF-α (*n* = 4–7) (**C**) and IL-1β (*n* = 3–7) (**D**) and the lesions levels of VEGF (*n* = 4) (**B**) were measured in the endometriosis mice with different treatments, as indicated by ELISA. * *p* < 0.05, *** *p* < 0.001 compared with the control group, ## *p* < 0.01 compared with the sham group. + *p* < 0.05, ++ *p* < 0.01 compared with the E2 group. C: control, S: sham, E2: β-estradiol, LFC: low dose of fucoidan, MFC: mid dose of fucoidan, HFC: high dose of fucoidan.

In order to investigate anti-angiogenesis and anti-inflammatory effects of FC in endometriotic mice, we collected the endometriotic lesions and serum to conduct an ELISA assay of VEGF and inflammatory cytokines. As demonstrated in Figure 5C,D, E2 treatment significantly increased VEGF, IL-1β, and IL-1β, and TNF-α of serum compared with the control group and sham group, respectively. The level of IL-1β of serum of LFC group was markedly lower than that of the E2 group. In addition, the levels of IL-1β and TNF-α in the serum of HFC were also significantly lower than that of the E2 group. As shown in Figure 5A,B, the levels of VEGF in the endometriotic lesions of the FC-treated groups (LFC, MFC, and HFC) were significantly lower than that of the E2 group, but differences in serum were not significant, showing that after FC, treatment can reverse E2-induced inflammatory and angiogenesis effects.

## 4. Discussion

The development of endometriosis includes the role of the intracellular production of estrogen, which is related to the aromatase activity. During estrogen metabolism, the related target genes such as aromatase, ER-α, and ER-β can modulate the inflammatory cytokine, angiogenesis, and apoptosis progression of endometriosis [35].

Estradiol contributes to different gynecological diseases [36]. In breast cancer, research has shown estradiol-induced EMT and tumor growth [37]. Endometriosis is also an estrogen-dependent disease and occurs during the reproductive age [34]. A previous study also shows that estradiol can increase EMT progression in the endometriosis cell line [33]. The pathology of endometriosis is the endometrial-like tissue that migrates to the outside of the uterus, EMT progression shows a critical role in endometriosis [34]. In endometriosis, there is a decrease in the expression of epithelial markers and an increase in the expression of mesenchymal markers [38].

Recently, researchers have discovered some medication therapy for endometriosis, such as levonorgestrel intrauterine systems (LNG-IUSs) and depot medroxyprogesterone acetate (DMPA). The randomized control trial proved that the LNG-IUSs and DMPA treatment can improve patient compliance and give satisfaction, but it still presents side effect. Other methods like vaginal rings and novel drug delivery were effective in the in vitro or animal study. However, there is still a lack of evidence and some are still in the early experimental development [26].

Natural products and traditional medicine are widely used to cure the disorder or prevent disease [27]. Curcumin, with its anti-inflammatory effect, can reduce the cytokine and chemokines to relieve pain and reduce inflammation [39]. The pathology of endometriosis is complicated, given the target of traditional herbal compounds, such as Alchemilla and allium sativum, which have anti-inflammation and anti-angiogenesis effects and can eliminate the progression of endometriosis [40]. Dietary flavone quercetin and genistein also show a benefit on endometriosis, by inhibiting the proliferation of endometriosis with the cell cycle arrest [32], and they can decrease ER-α expression and the antiangiogenics effect [41]. Fucoidan shows an anti-inflammatory effect in diabetes-induced renal fibrosis and hyperuricemia related renal damage [42,43] and has an anti-proliferation effect in gynecological cancer [29]. This indicates the potential effect of fucoidan on endometriosis progression.

Endometriosis in endometrial tissue shifted to adhesion and proliferation in other tissue [24]. In a breast cancer study, FC 1–2 mg/mL significantly inhibited breast cancer cell MDA-MB-231 proliferation [44]. In a leiomyoma study, FC 0.1–1 mg/mL significantly inhibited leiomyoma cell ELT-3 proliferation [45], showing the anti-proliferation ability of FC. In the present study, estrogen 1 nM increased the cell viability and cell number of endometriotic cells and combined with FC 0.5, 1 mg/mL, and significantly inhibited E2-induced cell proliferation.

Although endometriosis is a benign tumor, it has features similar to those of malignant tumors, including invasion and migration ability. A previous study indicated that FC 1–2 mg/mL inhibited breast cancer cell invasion and migration ability [44]. In a liver cancer study, FC 50–200 μg/mL significantly inhibited liver cancer cell HepG2 migration ability [46]. In the present study, 1 nM estrogen increased the cell migration ability of endometriotic cells, increased the protein expression of N-cadherin and Vimentin, and reduced E-cadherin. FC (0.25, 0.5, and 1 mg/mL) significantly reduced the protein expression of N-cadherin and Vimentin, increased E-cadherin, and inhibited cell migration ability. In an animal study, the results also indicated that FC affects anti-migration ability. The activation of estrogen receptor alpha (ER-α) can induce an increase in Vimentin and a decrease in E-cadherin protein expression, which induced the EMT program [47]. The current study indicates that FC can decrease the EMT progression by decreasing ER-α.

Endometriosis is an inflammatory disease caused by increased TNF-α and IL-1β, which reduce the protein expression of Bax and increase Bcl-2 to inhibit the apoptosis of endometriotic lesions [18]. A previous study indicated that FC inhibited inflammation and induced apoptosis of cancer cells. In a leiomyoma study, FC 0.5 mg/mL significantly promoted the apoptosis effect in leiomyoma cell ELT-3 [45]. In a breast cancer study, FC 6.25–25 μg/mL significantly promoted the apoptosis ability in breast cancer cell MDA-MB-231 [48]. In the current study’s in vivo experiment, the levels of TNF-α, IL-1β of serum and VEGF of endometriosis lesions significantly decreased in the FC groups at doses of 10, 50, and 150 mg/kg compared with the E2 group. In addition, treatment with FC, Bcl-2 was significantly elevated compared with the E2 group, and Bax significantly decreased in the endometriotic lesions. These results suggest that FC has an inhibitory effect on inflammation and promoted apoptosis in endometriotic lesions in mice.

Angiogenesis is an important factor in endometriosis and is essential to cancer lesion growth. A previous study indicated that inflammation would increase VEGF to promote the angiogenesis of endometriosis [19]. In a prostate cancer study, FC (500 μg/mL) significantly inhibited prostate cancer cell DU-145 angiogenesis [30]. The in vivo experiments verified that FC (10, 50, and150 mg/kg) significantly reduced the VEGF level and the protein expression of PCNA on endometriotic lesions, showing that FC can reduce the inflammatory effect, not only triggering the apoptosis ability but also specifically reducing the endometriosis lesion’s angiogenesis effect to effectively decrease the proliferation of endometriosis.

## 5. Conclusions

**Figure 6:**
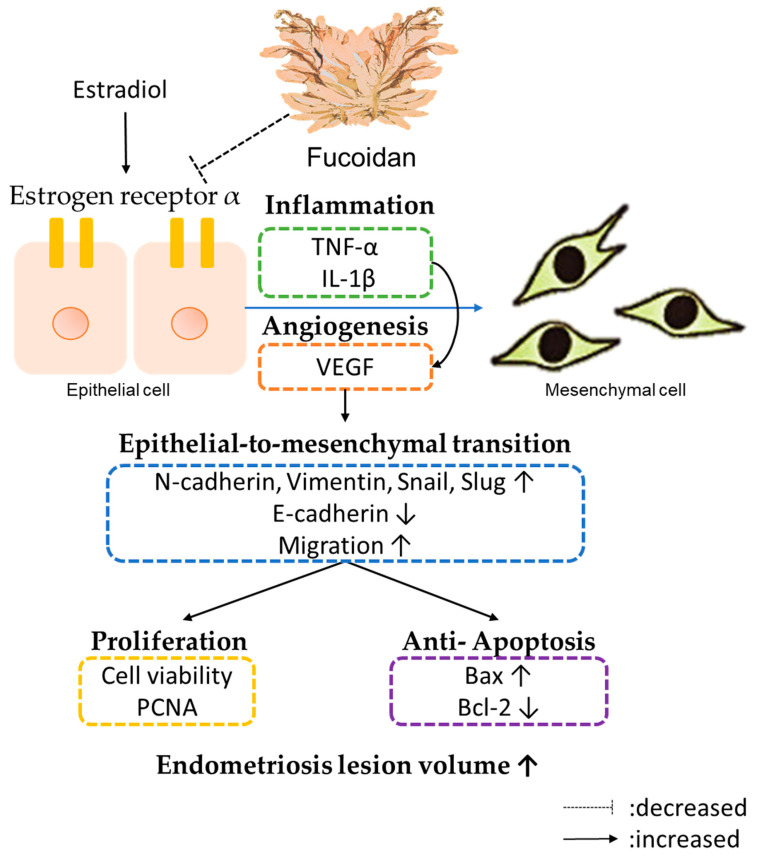
Summary of the mechanism of fucoidan against endometriosis. FC can effectively inhibit cell proliferation and migration of endometriosis cells by inhibiting EMT-related protein expression (N-cadherin, Vimentin, Snail and Slug). FC can also decrease the expression of the anti-apoptosis protein Bcl-2 and increase the expression of Bax, which suggests that FC-induced apoptosis is able to inhibit endometriosis progression. In addition, FC inhibited inflammation by regulating the levels of VEGF IL-1β and TNF-α and suppressed EMT in endometriosis mice

The current study’s results demonstrate that FC can effectively inhibit cell proliferation and cell migration of endometriosis cells by inhibiting EMT-related protein expression. FC can also decrease the expression of the anti-apoptosis protein Bcl-2 and increase the expression of Bax, which suggests that FC-induced apoptosis is able to inhibit endometriosis progression. In addition, FC inhibited inflammation by regulating the levels of VEGF IL-1β and TNF-α and suppressed EMT in endometriosis mice (Figure 6). Hence, FC has the potential to be a functional formula to improve endometriosis.

## References

[1] Bulun S.E. (2009). Endometriosis. N. Engl. J. Med..

[2] Matsuzaki S., Pouly J.L., Canis M. (2018). In vitro and in vivo effects of MK2206 and chloroquine combination therapy on endometriosis: Autophagy may be required for regrowth of endometriosis. Br. J. Pharmacol..

[3] Giudice L.C., Kao L.C. (2004). Endometriosis. Lancet (Lond. Engl.).

[4] Bulletti C., Coccia M.E., Battistoni S., Borini A. (2010). Endometriosis and infertility. J. Assist. Reprod. Genet..

[5] Wu R., Zhou W., Chen S., Shi Y., Su L., Zhu M., Chen Q., Chen Q. (2014). Lipoxin A 4 suppresses the development of endometriosis in an ALX receptor-dependent manner via the p38 MAPK pathway. Br. J. Pharmacol..

[6] Mogensen J.B., Kjar S.K., Mellemkjær L., Jensen A. (2016). Endometriosis and risks for ovarian, endometrial and breast cancers: A nationwide cohort study. Gynecol. Oncol..

[7] Sampson J.A. (1927). Peritoneal endometriosis due to the menstrual dissemination of endometrial tissue into the peritoneal cavity. Am. J. Obstet. Gynecol..

[8] Van Langendonckt. A., Francoise Casanas. R., Jacques. D. (2002). Oxidative stress and peritoneal endometriosis. Fertil. Steril..

[9] Nenicu A., Gu Y., Körbel C., Menger M.D., Laschke M.W. (2017). Combination therapy with telmisartan and parecoxib induces regression of endometriotic lesions. Br. J. Pharmacol..

[10] Mosher A., Tsoulis M.W., Lim J., Tan C., Agarwal S., Leyland N., Foster W. (2019). Melatonin activity and receptor expression in endometrial tissue and endometriosis. Hum. Reprod..

[11] El-Shennawy L., Dubrovskyi O., Kastrati I., Danes J.M., Zhang Y., Whiteley H.E., Creighton C.J., Frasor J. (2018). Coactivation of Estrogen Receptor and IKKβ Induces a Dormant Metastatic Phenotype in ER-Positive Breast Cancer. Cancer Res..

[12] Chen H., Malentacchi F., Fambrini M., Harrath A.H., Huang H., Petraglia F. (2020). Epigenetics of Estrogen and Progesterone Receptors in Endometriosis. Reprod. Sci. (Thousand Oaks Calif.).

[13] Paterni I., Granchi C., Katzenellenbogen J.A., Minutolo F. (2014). Estrogen receptors alpha (ERα) and beta (ERβ): Subtype-selective ligands and clinical potential. Steroids.

[14] Kulak J., Fischer C., Komm B., Taylor H.S. (2011). Treatment with bazedoxifene, a selective estrogen receptor modulator, causes regression of endometriosis in a mouse model. Endocrinology.

[15] Pluchino N., Mamillapalli R., Wenger J.M., Ramyead L., Drakopoulos P., Tille J.C., Taylor H.S. (2020). Estrogen receptor-α immunoreactivity predicts symptom severity and pain recurrence in deep endometriosis. Fertil. Steril..

[16] Symons L.K., Miller J.E., Kay V.R., Marks R.M., Liblik K., Koti M., Tayade C. (2018). The Immunopathophysiology of Endometriosis. Trends Mol. Med..

[17] Berkkanoglu M., Arici A. (2003). Immunology and endometriosis. Am. J. Reprod. Immunol..

[18] Kaur K.K., Allahbadia G. (2016). An Update on Pathophysiology and Medical Management of Endometriosis. Adv. Reprod. Sci..

[19] Kiriakidis S., Andreakos E., Monaco C., Foxwell B., Feldmann M., Paleolog E. (2003). VEGF expression in human macrophages is NF-κB-dependent: Studies using adenoviruses expressing the endogenous NF-κB inhibitor IκBα and a kinase-defective form of the IκB kinase 2. J. Cell Sci..

[20] Yang L., Cao Z., Yu B., Chai C. (2015). An in vivo mouse model of primary dysmenorrhea. Exp. Anim..

[21] Albertsen H.M., Ward K. (2017). Genes linked to endometriosis by GWAS are integral to cytoskeleton regulation and suggests that mesothelial barrier homeostasis is a factor in the pathogenesis of endometriosis. Reprod. Sci..

[22] Park S.-H., Cheung L.W.T., Wong A.S.T., Leung P.C.K. (2008). Estrogen regulates Snail and Slug in the down-regulation of E-cadherin and induces metastatic potential of ovarian cancer cells through estrogen receptor alpha. Mol. Endocrinol..

[23] Thiery J.P. (2002). Epithelial–mesenchymal transitions in tumour progression. Nat. Rev. Cancer.

[24] Bartley J., Julicher A., Hotz B., Mechsner S., Hotz H. (2014). Epithelial to mesenchymal transition (EMT) seems to be regulated differently in endometriosis and the endometrium. Arch. Gynecol. Obs..

[25] Nieto M.A. (2002). The snail superfamily of zinc-finger transcription factors. Nat. Rev. Mol. Cell Biol..

[26] Garzon S., Laganà A.S., Barra F., Casarin J., Cromi A., Raffaelli R., Uccella S., Franchi M., Ghezzi F., Ferrero S. (2020). Novel drug delivery methods for improving efficacy of endometriosis treatments. Expert Opin. Drug Deliv..

[27] Machairiotis N., Vasilakaki S., Kouroutou P. (2020). Natural products: Potential lead compounds for the treatment of endometriosis. Eur. J. Obstet. Gynecol. Reprod. Biol..

[28] Kan J., Hood M., Burns C., Scholten J., Chuang J., Tian F., Pan X., Du J., Gui M. (2017). A Novel Combination of Wheat Peptides and Fucoidan Attenuates Ethanol-Induced Gastric Mucosal Damage through Anti-Oxidant, Anti-Inflammatory, and Pro-Survival Mechanisms. Nutrients.

[29] van Weelden G., Bobiński M., Okła K., van Weelden W.J., Romano A., Pijnenborg J.M.A. (2019). Fucoidan Structure and Activity in Relation to Anti-Cancer Mechanisms. Mar. Drugs.

[30] Rui X., Pan H.F., Shao S.L., Xu X.M. (2017). Anti-tumor and anti-angiogenic effects of Fucoidan on prostate cancer: Possible JAK-STAT3 pathway. BMC Complement. Altern. Med..

[31] Fernando I.P.S., Dias M., Madusanka D.M.D., Han E.J., Kim M.J., Jeon Y.J., Lee K., Cheong S.H., Han Y.S., Park S.R. (2020). Human Keratinocyte UVB-Protective Effects of a Low Molecular Weight Fucoidan from Sargassum horneri Purified by Step Gradient Ethanol Precipitation. Antioxidants.

[32] Park S., Lim W., Bazer F.W., Whang K.Y., Song G. (2019). Quercetin inhibits proliferation of endometriosis regulating cyclin D1 and its target microRNAs in vitro and in vivo. J. Nutr. Biochem..

[33] Hsu Y.W., Chen H.Y., Chiang Y.F., Chang L.C., Lin P.H., Hsia S.M. (2020). The effects of isoliquiritigenin on endometriosis in vivo and in vitro study. Phytomedicine.

[34] Machairiotis N., Stylianaki A., Dryllis G., Zarogoulidis P., Kouroutou P., Tsiamis N., Katsikogiannis N., Sarika E., Courcoutsakis N., Tsiouda T. (2013). Extrapelvic endometriosis: A rare entity or an under diagnosed condition?. Diagn. Pathol..

[35] Laganà A.S., Garzon S., Götte M., Viganò P., Franchi M., Ghezzi F., Martin D.C. (2019). The Pathogenesis of Endometriosis: Molecular and Cell Biology Insights. Int. J. Mol. Sci..

[36] Xiong W., Zhang L., Liu H., Li N., Du Y., He H., Zhang Z., Liu Y. (2019). E(2) -mediated EMT by activation of β-catenin/Snail signalling during the development of ovarian endometriosis. J. Cell. Mol. Med..

[37] Wang K.H., Kao A.P., Lin T.C., Chang C.C., Kuo T.C. (2012). Promotion of epithelial-mesenchymal transition and tumor growth by 17β-estradiol in an ER(+)/HER2(+) cell line derived from human breast epithelial stem cells. Biotechnol. Appl. Biochem..

[38] Bilyk O., Coatham M., Jewer M., Postovit L.M. (2017). Epithelial-to-Mesenchymal Transition in the Female Reproductive Tract: From Normal Functioning to Disease Pathology. Front. Oncol..

[39] Fadin M., Nicoletti M.C., Pellizzato M., Accardi M., Baiett M.G. (2020). Effectiveness of the integration of quercetin, turmeric, and N-acetylcysteine in reducing inflammation and pain associated with endometriosis. In vitro and in vivo studies. Minerva Ginecol..

[40] Della Corte L., Noventa M., Ciebiera M., Magliarditi M., Sleiman Z., Karaman E., Catena U., Salvaggio C., Falzone G., Garzon S. (2020). Phytotherapy in endometriosis: An up-to-date review. J. Complement. Integr. Med..

[41] Sutrisno S., Aprina H., Simanungkalit H.M., Andriyani A., Barlianto W., Sujuti H., Santoso S., Dwijayasa P.M., Wahyuni E.S., Mustofa E. (2018). Genistein modulates the estrogen receptor and suppresses angiogenesis and inflammation in the murine model of peritoneal endometriosis. J. Tradit. Complement. Med..

[42] Yu W.C., Huang R.Y., Chou T.C. (2020). Oligo-Fucoidan Improves Diabetes-Induced Renal Fibrosis via Activation of Sirt-1, GLP-1R, and Nrf2/HO-1: An In Vitro and In Vivo Study. Nutrients.

[43] Chau Y.T., Chen H.Y., Lin P.H., Hsia S.M. (2019). Preventive Effects of Fucoidan and Fucoxanthin on Hyperuricemic Rats Induced by Potassium Oxonate. Mar. Drugs.

[44] Hsu W.J., Lin M.H., Kuo T.C., Chou C.M., Mi F.L., Cheng C.H., Lin C.W. (2020). Fucoidan from Laminaria japonica exerts antitumor effects on angiogenesis and micrometastasis in triple-negative breast cancer cells. Int. J. Biol. Macromol..

[45] Chen H.Y., Huang T.C., Lin L.C., Shieh T.M., Wu C.H., Wang K.L., Hong Y.H., Hsia S.M. (2018). Fucoidan Inhibits the Proliferation of Leiomyoma Cells and Decreases Extracellular Matrix-Associated Protein Expression. Cell. Physiol. Biochem..

[46] P A., K A., L S., M M., K M. (2019). Anticancer effect of fucoidan on cell proliferation, cell cycle progression, genetic damage and apoptotic cell death in HepG2 cancer cells. Toxicol. Rep..

[47] Di Zazzo E., Galasso G., Giovannelli P., Di Donato M., Bilancio A., Perillo B., Sinisi A.A., Migliaccio A., Castoria G. (2019). Estrogen Receptors in Epithelial-Mesenchymal Transition of Prostate Cancer. Cancers.

[48] Xue M., Ji X., Xue C., Liang H., Ge Y., He X., Zhang L., Bian K., Zhang L. (2017). Caspase-dependent and caspase-independent induction of apoptosis in breast cancer by fucoidan via the PI3K/AKT/GSK3β pathway in vivo and in vitro. Biomed. Pharmacother..

